# Vesicular Stomatitis Virus-Based Oncolytic Virotherapy: Recent Progress and Emerging Trends

**DOI:** 10.3390/curroncol32110627

**Published:** 2025-11-07

**Authors:** Cassandra Catacalos-Goad, Charlotte Johnstone, Valery Z. Grdzelishvili

**Affiliations:** Department of Biological Sciences, University of North Carolina at Charlotte, Charlotte, NC 28223, USA; ccatacal@charlotte.edu (C.C.-G.); cjohn449@charlotte.edu (C.J.)

**Keywords:** oncolytic virotherapy, cancer therapy, vesicular stomatitis virus, VSV, rhabdovirus, Maraba virus, Morreton virus, Jurona virus

## Abstract

Oncolytic virotherapy has emerged as a promising and innovative approach to cancer treatment, leveraging viruses that selectively replicate in tumor cells and cause their destruction (oncolysis), while simultaneously stimulating anti-tumor immune responses. Vesicular stomatitis virus (VSV), a prototypic rhabdovirus, is among the most versatile oncolytic virus platforms due to its favorable biological characteristics, including rapid replication and cell lysis, lack of pre-existing immunity in humans, and amenability to genetic engineering. Over the past decade, significant progress has been made in VSV-based oncolytic virotherapy. This review presents a comprehensive update on developments since our last review, emphasizing improvements in VSV safety, oncoselectivity, tumor-specific replication, direct oncolysis, and induction of antitumor immunity. By integrating recent applied discoveries with foundational knowledge, this review aims to guide ongoing efforts to advance VSV-based oncolytic virotherapy toward broader clinical translation and improved cancer patient outcomes. Additionally, we provide an overview of three closely related rhabdoviruses (Maraba, Morreton, and Jurona viruses) as emerging oncolytic platforms currently under preclinical and clinical investigation.

## Simple Summary

Cancer remains one of the leading causes of death worldwide, and many patients do not respond well to traditional treatments like chemotherapy or radiation. Oncolytic virotherapy is an innovative approach that uses viruses that selectively replicate in tumor cells and cause their destruction (oncolysis), while simultaneously stimulating anti-tumor immune responses. Vesicular stomatitis virus (VSV) has emerged as a promising candidate due to its strong oncolytic activities, ability to be safely engineered, and the absence of pre-existing immunity in humans. In recent years, researchers have made significant progress in improving the safety and effectiveness of VSV-based treatments, including strategies to help the virus better target tumors, avoid early virus clearance by the immune system, and stimulate strong anti-cancer immune responses. This review highlights the latest advances in VSV research and explores other closely related viruses, providing a foundation for future studies and the development of new cancer therapies.

## 1. Introduction

Despite rapid advances in understanding human biology, cancer remains a significant cause of morbidity and mortality worldwide [1]. Consequently, there is an urgent need to develop novel, highly effective therapeutic strategies to improve outcomes in cancer patients. Oncolytic virotherapy, which leverages the natural or genetically modified abilities of replication-competent viruses to selectively infect and replicate in cancer cells and cause their destruction (oncolysis), while sparing healthy tissue, offers an innovative and promising approach to cancer treatment. Historically, such potential of viruses was serendipitously observed when some cancer patients experiencing viral infections demonstrated spontaneous tumor regression, paving the way for intentional therapeutic application of viruses [2,3]. This early recognition, though limited by an incomplete understanding of viral biology and tumor immunology, laid the groundwork for modern oncolytic virotherapy. This approach has evolved significantly, progressing through multiple generations of viral engineering and clinical investigation, and now offers a highly promising and innovative strategy for cancer patients, particularly those resistant to conventional therapies [4,5].

Oncolytic viruses (OVs) mediate antitumor activity through both direct tumor cell lysis (“direct oncolysis”) and the activation of antitumor immune responses (“immunostimulation”). Direct oncolysis refers to the preferential replication of OVs within malignant cells, resulting in tumor cell lysis and the release of viral progeny that subsequently infect and destroy neighboring cancer cells [6,7]. This lytic process not only eradicates tumor cells but can also elicit potent anti-tumor immune responses, effectively turning immunologically “cold” tumors “hot” [5]. The field of oncolytic virotherapy is expanding rapidly, with increasing preclinical studies, human clinical trials, and approval of OV candidates. As of today, several OVs have been approved for therapeutic use in different regions. In the United States, the only FDA-approved OV is the HSV-1-based Imlygic (talimogene laherparepvec, or T-VEC) for the treatment of melanoma [4]. In Japan, the herpes simplex virus-based teserpaturev (also known as G47Δ) has been approved for malignant glioma [8]. In China, an adenovirus 5-based OV, Oncorine, is approved for certain cancers [9,10]. Additionally, RIGVIR, an enterovirus-based OV, was previously approved in Latvia, but since been withdrawn due to regulatory concerns [11]. These approvals highlight the increasing global recognition of the clinical potential of OV-based therapies for different malignancies.

**Figure 1:**
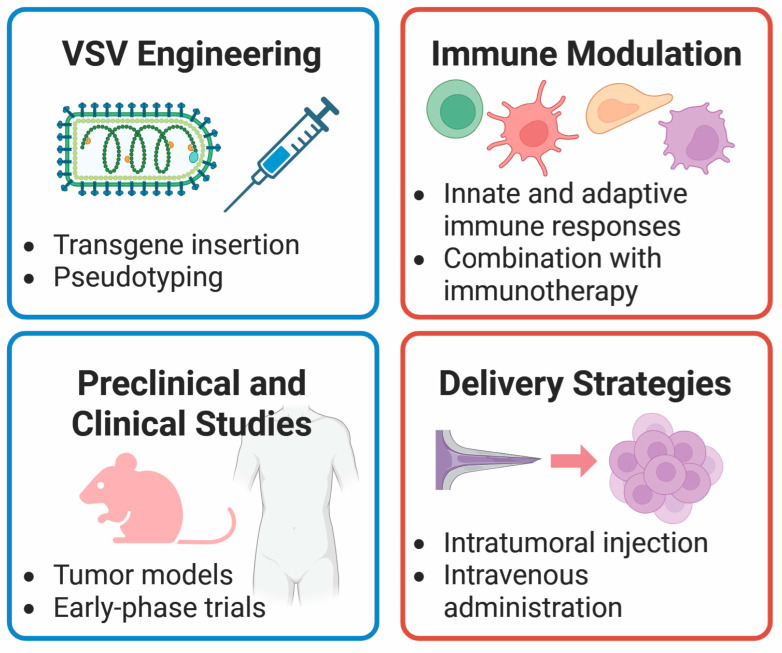
The schematic highlights significant advances in the VSV OV field over the past ten years. Top left quadrant: VSV engineering depicts genetic modifications to enhance safety, tumor selectivity, and therapeutic potency (e.g., gene order rearrangements, IFN-armed VSVs, glycoprotein mutations). Bottom left quadrant: Preclinical and clinical studies summarize translational progress from diverse cancer models to early-phase human trials, demonstrating safety, efficacy, and evolving therapeutic endpoints. Top right quadrant: Immune modulation illustrates how VSV therapy reshapes the tumor microenvironment, activates innate and adaptive immunity, and synergizes with immunotherapies. Bottom right quadrant: Delivery strategies outline approaches to optimize viral administration, including systemic versus i.t. routes, carrier cell-based delivery of VSV, and combination with chemical agents or biomaterials.

This review focuses on vesicular stomatitis virus (VSV) as a promising OV. VSV is a prototypic rhabdovirus (order *Mononegavirales*, family *Rhabdoviridae*, genus Vesiculovirus) with a small, 11-kb nonsegmented negative-strand RNA genome encoding five proteins (N, P, M, G, and L). VSV remains one of the promising OV platforms due to its favorable biological characteristics. These include the absence of pre-existing immunity in the human population (limiting premature clearance of viral infection), cytoplasmic replication without risk of genome integration or cell transformation, rapid amplification and cell lysis, very well-studied biology, and amenability to genetic engineering. In the 8 years since our last review of VSV-based oncolytic virotherapy [12], which followed our original comprehensive review of VSV as an OV [13], substantial progress has been made across safety, cancer cell targeting, oncolysis, immune engagement, and delivery strategies. Here, we present a comprehensive update on VSV-based oncolytic virotherapy research published in the last 8 years, highlighting new oncolytic VSV recombinants, combination strategies with conventional therapies and/or immune checkpoint inhibitors (ICIs), delivery innovations, and novel insights into targeting the tumor microenvironment (TME) (Figure 1). In addition, we provide an overview of three closely related rhabdoviruses (Maraba, Morreton, and Jurona viruses) as emerging oncolytic platforms currently under preclinical and clinical investigation. One of them, Maraba virus, is now advancing through early clinical trials in Canada (Clinical Trials: NCT02285816, NCT02879760).

This update aims to serve as a resource for both new and established investigators in the field, building on foundational knowledge while exploring the next frontier of VSV-based oncolytic virotherapy. For a more detailed account of earlier discoveries and foundational concepts, we refer readers to our original reviews and other comprehensive summaries on this topic [12,13,14,15].

## 2. Key Considerations for VSV Safety and Oncoselectivity

To ensure both efficacy and clinical applicability, oncolytic virotherapy requires the use of OVs that demonstrate two critical properties: safety and oncoselectivity. Safety ensures that the virus does not cause toxicity in the patient, particularly via infection and spread in normal tissues. Oncoselectivity refers to the virus’s ability to specifically or preferentially target cancer cells for infection, replication, and lysis, while sparing healthy, non-malignant cells. Ideally, this selectivity begins at the stage of viral attachment, with the OV recognizing and binding only to cell-surface receptors uniquely or predominantly expressed on cancer cells. If selective attachment to cancer cells cannot be achieved, oncoselectivity can still be ensured by restricting viral replication to tumor cells by exploiting their special cellular conditions that favor viral replication. These mechanisms include the presence of tumor-specific factors that support viral replication or the exploitation of deficiencies in antiviral restriction mechanisms, such as impaired innate immune responses, which are commonly found in cancer cells. This allows a greater proportion of the viral dose to reach its intended target, enhancing both potency and overall therapeutic outcome.

Wild-type (wt) VSV naturally infects livestock and rarely causes significant disease in humans, where infections are typically subclinical and limited to endemic regions of the Americas. VSV has a broad cellular tropism that is mainly attributable to its ability to exploit the low-density lipoprotein receptor (LDL-R) and other members of the LDL-R family as entry receptors [16,17], which are expressed on nearly all nucleated cells, including most tumor cells. Such widespread receptor expression contributes to the virus’s pantropism and limits its ability to distinguish between normal and tumor cells during virus attachment and entry. However, VSV pantropism also ensures that viral targeting of cancer cells is not limited by receptor availability. Additionally, this ubiquity makes it more difficult for tumor cells to evade infection by downregulating receptor expression [18]. VSV oncoselectivity does not rely on selective attachment to cancer cells; rather, it stems from the frequent deficiencies in type I interferon (IFN) signaling found in many tumors, a critical factor that allows VSV to preferentially replicate in cancer cells. However, wt VSV retains the capacity to suppress IFN responses through its matrix (M) protein in non-malignant cells, which can result in neurotoxicity, particularly in rodent and primate models [17,19,20,21]. To mitigate this challenge, multiple alternative (but often complementary) strategies have been used to sharpen VSV oncoselectivity and improve safety. Many studies employ rationally attenuated VSV recombinants encoding a mutant matrix (M) protein with ΔM51 or M51R mutation, which inhibit the abilities of VSV-M to suppress cellular IFN responses [22]. Alternatively, VSV recombinants are used that encode IFN transgenes, such as VSV-IFNβ, which both restrict replication in normal tissues and enhance tumor-specific immunity [23]. VSV-IFNβ-NIS is the most notable OV, which couples local type-I IFN production restricting replication in normal tissues with functional IFN signaling with NIS-based noninvasive imaging (with technetium-99m, iodine-123, etc.) or localized radioactive iodine (iodine-131) therapy. VSV-IFNβ-NIS is now being evaluated in hematologic and solid tumors in multiple early-phase clinical trials in the USA, including combinations with PD-1/CTLA-4 blockade (e.g., Clinical Trials NCT02923466, NCT03120624, NCT03017820, NCT03647163).

The creation of chimeric VSV recombinants encoding foreign glycoproteins has been central to efforts to reduce VSV attachment to normal cells (“detargeting”) and/or improve attachment to tumor cells (“retargeting”). For example, both detargeting and retargeting have been achieved in VSVs engineered to engage tumor-restricted receptors (e.g., HER2-directed chimeric glycoproteins or adapter systems), which enhanced tumor tropism and reduced off-tumor infection [24]. Alternatively, microRNA-guided restriction of VSV replication to tumor cells has gained traction, whereby recombinant VSVs incorporate miRNA target sites to restrict viral replication in normal tissues expressing those miRNAs, thereby enhancing tumor selectivity without compromising oncolytic potency [25]. Collectively, these advances described below are converging to deliver safer, more tumor-selective VSV platforms. These topics are examined below, with a focus on oncolytic VSV research published in the last 8 years.

## 3. Chimeric VSV Recombinants to Reduce Viral Attachment to Normal Cells

Safety has been a central concern in the clinical translation of VSV-based oncolytic virotherapy, particularly given the virus’s pantropic nature and potential neurotoxicity. In this section, we describe strategies to prevent VSV attachment to normal cells and redirect it toward cancer cells. One foundational approach has been glycoprotein swapping in chimeric VSVs. It should be noted that, while the terms “chimeric” and “pseudotyped” are often used inter-changeably in the literature, in this review, we will use the traditional term “chimeric” for replication-competent VSV recombinants in which the VSV-G gene is replaced with a foreign envelope gene, enabling both entry and subsequent rounds of viral replication. In contrast, the term “pseudotyped” will be used for replication-incompetent vectors in which the original envelope gene is deleted from viral genome (which do not encode a foreign envelope gene) and a foreign envelope protein is incorporated into viral particle to allow a single round of entry. A comprehensive summary of the various chimeric VSV constructs, including their applications, is provided in Table 1 at the end of this section, and the extended list in present in the Supplemental Table S1 that shows the details on genetic modifications, targeted mechanisms of action, therapeutic applications, and associated cancer models.

An earlier study engineered a chimeric virus by replacing its native glycoprotein (VSV-G) with that of lymphocytic choriomeningitis virus (LCMV-GP), creating a chimeric construct termed VSV-GP [26,27]. In vitro, VSV-GP-infected glioma cell lines were infected at least as efficiently as with wt VSV, while sparing primary neurons. This reduction in neurotropism occurs because the LCMV glycoprotein redirects viral entry to cells expressing α-dystroglycan. This receptor is mainly inaccessible or non-functional in neurons, thereby preventing efficient infection of neural tissue. In glioma-bearing mice, systemic administration of VSV-GP achieved robust tumor regression without observable neurotoxicity, even at doses lethal for wt VSV. Moreover, VSV-GP enabled evasion of pre-existing anti-VSV antibodies, allowing safe repeat dosing without loss of oncolytic efficacy. This work established a critical proof-of-concept for enhancing VSV safety through receptor-targeted viral envelope engineering and has informed the design of subsequent chimeric VSV platforms aimed at broadening therapeutic windows and enabling systemic administration in humans. More recent studies have advanced VSV-GP into first-in-human testing and detailed pharmacokinetics and biodistribution analyses, with an expected more favorable safety profile for systemic use [28,29,30]. VSV-GP has entered human clinical testing, including a Phase I dose-escalation study, to assess its safety, tolerability, and preliminary efficacy as both a monotherapy and in combination with other treatments (Clinical Trial NCT04046445). Advancements in genetic engineering have enabled the development of VSV-GP variants expressing immune-modulatory cytokines, such as interleukin-12 (IL-12), to enhance antitumor immunity [31]. Additionally, improvements in manufacturing processes, including perfusion-based systems, have been implemented to increase viral production yields and ensure consistent quality for clinical applications [32,33]. Current research continues to explore the immunomodulation of VSV-GP as a monotherapy [34] or in combination with ICIs and other immunotherapies/cancer vaccines [35,36,37], and will be discussed more in the appropriate section. In lung cancer, VSV-GP showed potent lytic activity in IFN receptor-deficient LLC1 tumors, where direct viral oncolysis dominated therapeutic outcomes, independent of CD8^+^ T cells [38]. In melanoma models, combination therapy with VSV-GP and dendritic cell (DC) vaccines increased CD8^+^ T cell infiltration, reduced regulatory T cells, and enhanced survival, indicating that immune modulation, rather than direct lysis, contributed to efficacy [37]. Additionally, VSV-ΔM51-GP, an attenuated VSV-GP variant, preferentially infected immature DC and induced maturation with minimal cytotoxicity, highlighting the importance of viral glycoprotein selection and attenuation in modulating host immune responses [39]. This platform can be used for both oncolytic therapy and vaccination (prophylaxis) strategies. VSV-GP expressing HPV16 antigens (E2, E6, and E7) showed both preventive and therapeutic effects against HPV in TC-1 tumor models, and first position of the vaccine antigen in VSV genome was shown to be superior to fifth position [40].

Another promising chimeric VSV is rVSV-NDV, in which VSV-G is replaced with the hemagglutinin-neuraminidase (HN) and modified fusion (F) proteins from Newcastle disease virus (NDV, a paramyxovirus). rVSV-NDV combines VSV’s rapid replication with NDV’s fusogenic properties, enabling efficient tumor spread and immunogenic cell death. In preclinical hepatocellular carcinoma (HCC) models, rVSV-NDV produced prolonged survival with reduced neuro- and hepatotoxicity, establishing a foundation for safer chimeric VSV-based therapies [41]. VSV-NDV was later evaluated in melanoma models, demonstrating its ability to elicit robust anti-tumor immune responses. In a B16 melanoma model, intratumoral (i.t.) VSV-NDV injection induced strong innate and adaptive immunity, with NK cell activation, DC maturation, and CD8^+^ T cells, which were shown to infiltrate into both treated and distant tumors and were essential for tumor control [42]. Building on this, another study showed that VSV-NDV synergizes with immune checkpoint blockade (anti-CTLA-4), enhancing tumor-specific T cell activation via tumor RIG-I signaling, which was critical for the combined therapeutic effect [43]. This will be further elaborated in the appropriate immune modulation section of this review.

Other chimeric VSV constructs include VMG and LASV-VSV. VMG, in which VSV-G is replaced with Morreton virus glycoprotein, showed broad oncolytic activity in human, murine, and canine sarcoma lines. In vivo, a single i.t. VMG dose completely inhibited Ewing sarcoma xenografts, whereas in immunocompetent fibrosarcoma models, VMG induced substantial CD8^+^ T cell infiltration despite limited tumor regression, demonstrating both safety and immune engagement [44]. Similarly, LASV-VSV, in which the VSV glycoprotein was replaced with Lassa virus glycoprotein, targeted human and murine ovarian cancers effectively, showing oncolysis and systemic anti-tumor immunity in immunocompetent mice, as well as improved safety for brain exposure [45].

A key obstacle in VSV-based therapy for brain tumors is achieving adequate tumor clearance while avoiding damage to healthy neural tissue by this potentially neurotropic virus. Anthony van den Pol’s group made significant contributions in this area, initially identifying parvovirus LuIII as particularly effective among several parvoviruses tested, noting its relative resistance to IFN-mediated inhibition, which can limit antiviral activity in the brain [46]. Of particular relevance to VSV-based approaches, this group demonstrated that replacing the native VSV-G with glycoproteins from heterologous viruses reduced neurotoxicity without compromising the ability of VSV to kill tumor cells [47]. They further expanded on this work using chimeric VSV-EBOV encoding Ebola virus glycoprotein to find that it selectively eliminated glioblastoma (GMB) tumors while sparing normal brain tissue, attributing this observed oncoselectivity to its mucin-like domain, demonstrating the critical role of glycoprotein modifications in improving selectivity and safety [48].

**Table 1:** Chimeric VSVs.

Virus	Foreign Envelope	Cancer Target	Model	Key Findings	Primary Effect	Stage of Development	Ref.
rVSV-NDV	NDV HN + F proteins	Hepatocellular carcinoma (HCC)	Mouse preclinical	Efficient tumor spread, immunogenic cell death, prolonged survival, and reduced neuro- and hepatotoxicity.	Lytic & immune	Pre-clinical	[41]
rVSV-NDV	NDV HN + F proteins	Melanoma (B16)	Syngeneic, dual-flank tumors	Strong innate & adaptive immunity, NK & CD8^+^ T cell activation, systemic anti-tumor response; CD8^+^ T cells essential.	Immune	Pre-clinical	[42]
VSV-NDV + anti-CTLA-4	NDV HN + F proteins	Melanoma	Syngeneic	Synergistic with checkpoint blockade, tumor-specific T cell activation is dependent on tumor RIG-I signaling.	Immune	Pre-clinical	[43]
VSV-GP	LCMV GP	HPV+ tumors (TC-1)	Mouse	Prophylactic and therapeutic benefits: the position of the antigen in the genome affects expression and immune activation.	Immune	Pre-clinical	[40]
VSV-GP	LCMV GP	Lung carcinoma (LLC1)	Syngeneic	Direct lytic activity dominates tumor regression, with minimal contribution from adaptive immunity.	Lytic	Pre-clinical	[38]
VSV-GP + DC vaccine	LCMV GP	Melanoma	Mouse	Enhanced survival via immune modulation, increased CD8^+^ T cell infiltration, reduced Tregs, and minimal direct lysis.	Immune	Pre-clinical	[37]
VSV-dM51-GP	LCMV GP, attenuated	N/A (immune activation studies)	Mouse & human DCs	Preferentially infects immature DCs, induces maturation, and reduces cytotoxicity.	Immune	Pre-clinical	[39]
VMG	Morreton virus GP	Sarcoma (Ewing, fibrosarcoma)	Mouse xenografts & immunocompetent models	Broad oncolysis in vitro, complete xenograft inhibition, and increased CD8^+^ T cell infiltration in immunocompetent models.	Lytic & immune	Pre-clinical	[44]
LASV-VSV	Lassa virus GP	Ovarian cancer	Mouse, immunocompetent & immunocompromised	Efficient oncolysis, systemic anti-tumor immunity, and minimal neurotoxicity.	Lytic & immune	Pre-clinical	[45]
VSV-EBOV	Ebola GP with mucin-like domain	Glioblastoma	SCID mice	Selective tumor killing, minimal normal brain infection, enhanced oncolytic efficacy, and safety.	Lytic	Pre-clinical	[48]
VSV-GP (BI 1831169)	LCMV GP (glycoprotein)	Advanced, metastatic, or relapsed solid tumors	Patients; monotherapy and combination with anti-PD-1	First-in-human study designed to determine safety, tolerability and MTD/RP2D for monotherapy and for combination with ezabenlimab. Evaluates IT, IV, and combined dosing routes.	Lytic & immune	Early clinical (Phase I)	ClinicalTrials.gov NCT0515533, [28]

## 4. Enhancing VSV Tumor Tropism via Experimental Adaptation

Experimental adaptation of VSV to specific cancer cell types aims to improve viral replication and oncolytic effectiveness in partially resistant tumors. This method involves serial passaging of VSV in cancer cell lines to select viral variants with improved oncolytic potency. Our recent study used a directed viral evolution approach to create new oncolytic VSVs with better replication in pancreatic ductal adenocarcinoma (PDAC) cell lines. We utilized two previously described oncolytic VSV recombinants, VSV-p53wt and VSV-p53-CC, which encode a VSV matrix protein with a ΔM51 mutation and a functional human tumor suppressor p53 fused to a fluorescent reporter protein eqFP650. Each virus was passaged 32 times on either the SUIT-2 (moderately resistant to VSV) or MIA PaCa-2 (highly permissive to VSV) human PDAC cell lines. While no phenotypic changes occurred in MIA PaCa-2-passaged viruses, SUIT-2-passaged variants showed improved replication in SUIT-2 and AsPC-1 (another moderately resistant PDAC cell line), while remaining attenuated in non-malignant cells. Both acquired identical VSV-G mutations (K174E and E238K) that improved viral attachment and replication. No alterations occurred in the M-ΔM51, p53, or eqFP650 regions, which shows the long-term stability of these complex VSV recombinants carrying large transgenes [49].

Another study used experimental viral evolution by serially passaging VSV in breast cancer cell monolayers, spheroids, and tissue explants [50]. This allowed the virus to adapt to the TME and select for variants with improved virus replication and oncolytic potential under different cellular contexts. The authors identified specific mutations in the VSV genome, particularly in VSV-G, that enhanced viral attachment and replication in resistant tumor cells. Adapted viruses maintained oncolytic selectivity, showing improved cytotoxicity in cancer cells while remaining attenuated in non-malignant cells. Additionally, adaptation outcomes varied across cellular models (monolayers, spheroids, and tissue explants), highlighting the importance of tumor architecture in shaping viral evolution.

These findings underscore the evolutionary plasticity of VSV and its potential for developing personalized oncolytic virotherapies tailored to specific tumor types or even individual patients. By leveraging VSV’s adaptability through experimental evolution, researchers can generate variants with enhanced replication, tumor selectivity, and oncolytic potency, particularly in cancers resistant to standard therapies. This approach also provides valuable insights into viral–host interactions, mechanisms of antiviral resistance, and determinants of viral tropism, which can inform the rational design of safer and more effective OVs.

## 5. Improving Preferential Replication of VSV in Tumor Cells

In the previous section, we explored strategies to prevent VSV attachment to normal cells and redirect it toward cancer cells. In this section, we focus on strategies to improve tumor-selective replication of VSV by improving its selective replication in cancer cells. As noted earlier, the oncoselectivity of VSV encoding the VSV-G protein does not primarily depend on selective attachment to tumor cells, as it uses LDL-R, which is expressed on nearly all nucleated cells. Instead, it relies mainly on the frequent deficiencies in type I IFN signaling in many cancers, a key factor that enables VSV to replicate preferentially in malignant cells. The approaches discussed below primarily aim to limit VSV replication in normal cells while preserving, or even enhancing, its replication in tumor cells. These approaches include (1) attenuating VSV by disrupting regular gene order, (2) VSV recombinants encoding M protein with a methionine mutation at position 51, (3) VSV recombinants encoding IFN transgenes, (4) combining VSV with JAK inhibitors, (5) combining VSV with epigenetic modulators, (6) combining VSV with metabolic rewiring, and (7) VSV encoding vaccinia virus genes evading antiviral immune responses.

### 5.1. Attenuating VSV by Disrupting Normal Gene Order

By exploiting the natural transcriptional gradient of VSV, in which genes closer to the 3′ end of the VSV genome are transcribed more efficiently, researchers can rationally attenuate the virus, thereby reducing overall replication and pathogenicity without completely compromising anticancer therapeutic activity. Early studies in the late 1990s and early 2000s demonstrated that systematic rearrangement of genes such as N, P, and L produced stepwise attenuation, creating a platform for exploring viral biology, oncolytic potential, and neurotoxicity thresholds [51,52,53,54]. These pioneering experiments helped establish gene order as a key determinant of VSV virulence and informed the design of subsequent attenuated viral strains. A recent study has demonstrated that VSV gene order manipulation, such as inserting foreign genes at position 1 (before the VSV nucleocapsid (N) gene), reduced neurotoxicity while maintaining oncolytic potency in preclinical CNS tumor models [55].

However, the relative lack of recent studies focusing on gene order-based attenuation reflects both the scientific maturation of the approach and evolving research priorities. Modern VSV engineering favors more precise and tunable strategies, such as inserting interferon genes (e.g., IFN-β), introducing targeted point mutations like MΔ51, or incorporating tissue-specific microRNA target sites, to attenuate viral replication while preserving oncolytic potency. These methods allow fine control over viral kinetics, immune activation, and tissue specificity, which gene order rearrangement alone cannot achieve as effectively. Furthermore, extensive attenuation via gene rearrangement can significantly reduce viral fitness, potentially limiting efficacy in oncolytic applications or vaccine platforms that require robust replication in target cells. As a result, gene order manipulation has largely been supplanted by these more versatile and clinically adaptable genetic engineering strategies.

### 5.2. VSV Recombinants Encoding M Protein with a Methionine Mutation at Position 51

As indicated in the Introduction, wt VSV retains the capacity to suppress IFN responses through its matrix (M) protein, which can result in neurotoxicity, particularly in rodent and primate models [17,19,20,21]. One of the most commonly used approaches to rationally attenuate replication of VSV in healthy tissues is to use VSV-ΔM51 or VSV-M51R mutants that encode a mutant M protein [22]. These mutations impair the ability of the M protein to bind the Rae1-Nup98 mRNA export complex, thereby preventing the suppression of host gene expression (including expression of antiviral genes) in normal cells and enhancing the overall safety profile [56]. The VSV-ΔM51-based OVs are still used extensively in the field, and we refer readers to our previous comprehensive reviews for older studies exploiting them to improve VSV-based oncolytic virotherapy [12,13].

### 5.3. VSV Recombinants Encoding IFN Transgenes

VSV recombinants engineered to express type I, type II, or type III IFNs were developed to enhance tumor selectivity, limit neurotoxicity, and simultaneously stimulate antitumor immunity. Among these, type I IFNs, especially IFN-β, have emerged as one of the most clinically advanced strategies. The rationale is twofold: first, IFN-β can restrict viral replication in nonmalignant tissues, reducing off-target toxicity (especially neurotoxicity), by producing large amounts of IFNs by VSV-infected cells; second, the secreted IFNs and subsequent immunogenicity can enhance antitumor immune responses by promoting DC activation and cytotoxic T cell priming.

A recent systematic review of preclinical data demonstrated that VSV-IFNβ consistently reduces neurotoxicity while maintaining robust antitumor activity across various cancer models, including melanoma, hepatocellular carcinoma, and multiple myeloma [57]. Durham et al. showed that intravenous administration of VSV-IFNβ induced durable systemic control of multiple myeloma in immunocompetent mice. This effect was attributed to tumor-restricted IFN-β expression, which limited off-target viral replication and improved tolerability while promoting T cell–mediated immunity [58].

Beyond its direct immunostimulatory effects, IFN-β can also modulate the tumor microenvironment (TME) to enhance the efficacy of combination therapies. El Sayes et al. demonstrated that transient blockade of the type I IFN receptor before VSV-IFNβ treatment prevented the virus-induced upregulation of PD-L1, a primary immune checkpoint ligand. This approach sustained cytotoxic T cell activity, mitigated a key immunosuppressive feedback loop, and ultimately improved the therapeutic efficacy of VSV [59]. An innovative example of adaptive oncolytic virotherapy is a recent study that employed a sequential “trap-and-ambush” strategy [60]. An initial VSV-IFNβ variant drove tumor escape via a predictable CSDE1 (Cold Shock Domain-containing E1) mutation (CSDE1^P5S^) that enabled tumors to evade oncolytic virotherapy. However, when tumors develop this mutation, they not only become resistant to the OV but also produce a new neoantigen that can be targeted therapeutically. This was exploited by using a second treatment with an adapted VSV-IFNβP/M construct that selectively targeted the resistant clones. This two-step approach eliminated resistant tumor populations, cured approximately 50% of established B16 melanomas, and generated neoantigen-specific immune responses against CSDE1^P5S^ as well as TYRP2, a melanocyte differentiation antigen that serves as a shared self/tumor-associated antigen, indicating the induction of both neoantigen- and self-antigen-directed immunity. Importantly, combining this strategy with inhibition of the CD200 activation receptor ligand (CD200AR-L) re-sensitized tumors to checkpoint blockade therapy, transforming previously refractory models into responders [60].

While the majority of VSV research has focused on IFN-β, there is growing interest in leveraging type III IFNs (IFN-λ) to improve safety and immune modulation further. Type III IFNs signal through a distinct receptor (IFNLR1/IL10R2), which is more selectively expressed on epithelial and some tumor cells, potentially enabling more targeted modulation of the TME. Guayasamin et al. explored this concept by engineering VSV to express murine IFNL2 (encodes IFN-λ2, also known as IL-28A), inserting the cytokine gene into either the first (VSV28.1) or fifth (VSV28.5) position of the viral genome [61]. Both constructs were significantly attenuated in healthy tissues in vitro and in vivo but retained the capacity to induce virus-specific T cell and antiviral antibody responses in mice. These findings established proof of concept that IFN-λ can enhance the safety profile of VSV while preserving immunogenicity, particularly in vaccine contexts. However, the translational potential of VSV encoding IFN-λ remains uncertain. The study only evaluated murine IFN-λ2, leaving open questions about the efficacy and safety of human IFN-λ isoforms. Furthermore, the excessive attenuation, including at tumor sites, observed in both VSV28.1 and VSV28.5 may limit therapeutic efficacy in cancer models that require i.t. delivery for robust replication and effective direct oncolysis. The study was also limited by its focus on prophylactic vaccine applications rather than therapeutic tumor models, and by the lack of long-term studies to assess durability and safety in immunocompromised settings. Moreover, IFN-λ can exert opposing effects depending on context. While IFN-λ has been shown to have tumor-suppressive roles, its endogenous expression has also been linked to poor prognosis in cancer patients. Specifically, the human genes IFNL2 (which encodes IFN-λ2) and IFNL3 (which encodes IFN-λ3) serve as independent prognostic markers, and their expression can influence tumor progression through the JAK-STAT signaling pathway [62]. Abnormalities in IFN-λ genes are associated with alterations in immune checkpoints and immune cell infiltration, which, in turn, affect cancer- and immune-related pathways [62]. Understanding and balancing this duality will be critical for the future development of VSVs incorporating IFN-λ.

Beyond IFN-β, IFN-λ2, and IFN-λ3, other interferons, including type I IFN-α subtypes and type II IFN-γ, are also being investigated for their potential to enhance VSV-based therapy further. IFN-α shares many signaling properties with IFN-β but consists of multiple subtypes that may offer finer control over antiviral and immune-stimulatory responses. Select IFN-α variants have demonstrated synergy with VSV in preclinical models, promoting tumor cell apoptosis and enhancing antigen presentation to bolster T cell-mediated immunity [59,63,64]. Similarly, IFN-γ, a type II interferon, plays a central role in shaping adaptive immunity by activating macrophages, promoting Th1 polarization, and increasing MHC class I expression on tumor cells. Incorporating IFN-γ into VSV vectors can transform immunologically “cold” tumors into “hot” ones, making them more responsive to ICIs [65].

Despite their promise, all IFN transgenes pose challenges when used in OV platforms. Their potent antiviral effects may inhibit viral replication and spread in the tumor tissues, undermining the direct cytolytic component of VSV therapy. This is particularly important for tumors retaining at least partial IFN-dependent antiviral signaling. Therefore, careful dosing, spatial targeting, or inducible expression systems may be required to maximize therapeutic benefit while minimizing viral clearance. Future research should focus on optimizing these alternative IFN payloads to achieve a balance between stimulation of antitumor immunity and viral fitness in malignant and nonmalignant tissues.

### 5.4. Combining VSV with JAK Inhibitors

Some tumor types exhibit innate or acquired resistance to VSV (and other OVs) due to intact or partially functional IFN signaling. The JAK/STAT pathway is a central mediator of the IFN response, driving the expression of antiviral IFN-stimulated genes (ISGs) that restrict viral replication and spread. In many cancers, including pancreatic, ovarian, and lung cancers, this pathway remains at least partially functional despite tumor immune evasion strategies, creating a barrier to effective VSV replication and virus-mediated oncolysis. Pharmacologic inhibition of JAK/STAT signaling, therefore, represents a promising approach to sensitize resistant tumors to VSV by transiently suppressing antiviral defenses and increasing viral replication.

Our laboratory demonstrated the role of IFN signaling in VSV resistance in pancreatic cancer. We investigated why certain pancreatic ductal adenocarcinoma (PDAC) cell lines are resistant to VSV, and found that moderately-resistant PDACs rapidly upregulated type I IFNs following VSV exposure, leading to robust induction of ISGs and inhibition of viral replication [66,67]. In contrast, permissive PDAC cells exhibited defective IFN signaling, allowing productive viral infection and oncolysis. Surprisingly, we found that the most resistant PDAC cell lines not only induced expression of antiviral ISGs in response to viral infection but also displayed constitutive high-level expression of ISGs [66,68]. In another study, we identified 4 ISGs (MX1, EPSTI1, XAF1, and GBP1) that are constitutively co-expressed in VSV-resistant, but not in VSV-permissive PDACs, thus serving as potential biomarkers to predict OV therapy success [68]. Importantly, blocking IFN signaling using JAK inhibitors (e.g., FDA-approved JAK1/2 inhibitor ruxolitinib), which directly blocks JAK1/2 to prevent STAT1/2 activation downstream of type I IFN receptors, restored VSV susceptibility in otherwise resistant PDAC cells, establishing antiviral signaling as a key determinant of therapeutic response [66,69]. Our later study demonstrated that combining ruxolitinib with cationic polymers, which enhance viral attachment, could simultaneously overcome multiple resistance mechanisms in PDAC [70]. This combination therapy allowed VSV to bypass both extracellular barriers to infection and intracellular antiviral signaling, leading to robust viral replication and tumor cell death in resistant PDAC cells. These findings suggest that multi-pronged approaches targeting both the tumor cell surface and intracellular IFN pathways may be required for maximal sensitization in highly refractory cancers.

The therapeutic potential of JAK/STAT inhibition extends beyond PDAC. In ovarian cancer, which also exhibits heterogeneous responses to VSV, researchers tested ruxolitinib alone and in combination with VSV [71]. VSV monotherapy induced potent oncolytic activity in some ovarian cancer models, but in tumors with high IFN activity, combination treatment with ruxolitinib dramatically enhanced viral replication and tumor regression. These results indicate that JAK inhibition can expand the therapeutic reach of VSV by overcoming innate resistance in otherwise non-responsive tumors to VSV. Similarly, in non-small cell lung cancer (NSCLC), ruxolitinib sensitized resistant NSCLC cell lines to VSV-based oncolytic virotherapy, leading to greater viral spread and tumor cell killing [72]. This effect was linked to suppression of ISGs and IFN-stimulated antiviral signaling, further supporting the broad applicability of JAK/STAT targeting across cancer types.

Collectively, these studies establish the JAK/STAT pathway as a central barrier to VSV therapy and highlight ruxolitinib as a clinically relevant agent to overcome this barrier. By transiently suppressing tumor-intrinsic antiviral responses, JAK inhibitors enable more efficient viral replication and spread, converting resistant tumors into permissive targets for oncolytic virotherapy. Importantly, this approach is already translatable to the clinic, given FDA approval and numerous ongoing clinical trials of ruxolitinib for myeloproliferative disorders, along with its well-characterized safety profile (Clinical Trials NCT06034002, NCT01348490, NCT03144687, NCT02267278, NCT01375140, NCT06291987, NCT02076191, NCT00726232, NCT00952289). Moving forward, key considerations will include optimizing timing and dosing to avoid systemic immunosuppression, identifying biomarkers of resistance (e.g., baseline ISG expression), and exploring combination regimens with other sensitizing agents such as cationic polymers or ICIs. These strategies hold promise for unlocking the full therapeutic potential of VSV and broadening its use across a range of IFN-competent tumors.

### 5.5. Combining VSV with Epigenetic Modulators

Epigenetic regulation plays a critical role in determining how cancer cells respond to OVs, including VSV. Cellular epigenetic mechanisms, such as histone modifications and chromatin remodeling, can influence antiviral pathways, innate immune signaling, and tumor permissiveness to OV replication. Therefore, pharmacologic modulation of epigenetic pathways has emerged as a promising strategy to sensitize resistant tumors to OV-mediated oncolysis.

An example of a key epigenetic regulator implicated in VSV resistance is SIRT1, a class III histone deacetylase involved in chromatin remodeling and stress response regulation. A study demonstrated that SIRT1 levels directly influence the sensitivity of prostate cancer cells to VSV infection [73]. Using prostate cancer cell lines with varying susceptibility to VSV-mediated killing, the researchers manipulated SIRT1 expression through genetic knockdown and pharmacologic inhibition. Cells with high SIRT1 expression were significantly more resistant to VSV oncolysis, while SIRT1 suppression increased viral replication and cell death. Mechanistically, SIRT1 promoted the expression of antiviral ISGs and enhanced the host innate immune response against VSV. These findings identify SIRT1 as a negative regulator of VSV therapy and suggest that SIRT1 inhibition could be an effective strategy to increase VSV efficacy, particularly in prostate tumors with strong intrinsic antiviral defenses.

Building on this, another study identified two additional epigenetic factors, MAP3K7 and CHD1, as novel mediators of resistance to oncolytic VSV in prostate cancer [74]. Through a genome-wide CRISPR/Cas9 loss-of-function screen, the authors systematically interrogated genes involved in VSV sensitivity. They found that loss of either MAP3K7, a kinase involved in inflammatory signaling, or CHD1, a chromatin-remodeling factor, rendered previously resistant prostate cancer cells highly susceptible to VSV infection and oncolysis. Mechanistic studies revealed that these genes control antiviral signaling pathways and transcriptional programs that limit VSV replication. Notably, prostate cancer is frequently characterized by CHD1 loss, which may explain why certain tumors are inherently permissive to VSV, while those with intact CHD1 or MAP3K7 exhibit resistance. These results highlight the potential of epigenetic profiling to predict tumor responses to VSV and guide patient selection for VSV-based therapies.

Histone deacetylase inhibitors (HDACis) have also been shown to synergize with other OVs by dampening antiviral responses. A study found that HDACi treatment enhanced the oncolytic activity of an engineered oncolytic parainfluenza virus in cancer cells [75]. HDAC inhibition increased viral replication and potentiated tumor cell killing, while also blocking IFN-β production, a key component of the host antiviral defense. While this study focused on parainfluenza virus, the same principle could apply to VSV and other OVs that are highly sensitive to IFN-mediated antiviral responses.

Collectively, these studies illustrate the dual potential of epigenetic modulation in VSV-based cancer therapy. On one hand, tumor-intrinsic epigenetic factors such as SIRT1, CHD1, and MAP3K7 can determine whether a tumor is permissive or resistant to VSV infection, making them promising biomarkers for patient stratification. On the other hand, pharmacologic interventions such as HDACi can actively reshape the tumor epigenetic landscape to suppress antiviral defenses and enhance OV replication. Thus, integrating epigenetic modulators with VSV therapy offers a promising strategy to overcome resistance, expand the pool of responsive tumors, and improve therapeutic outcomes.

### 5.6. Combining VSV with Metabolic Rewiring

Metabolic reprogramming is a hallmark of cancer, enabling tumor cells to sustain uncontrolled proliferation, resist cell death, and adapt to hostile microenvironments. OVs exploit many of the same metabolic pathways as tumor cells to support their replication, creating a dynamic interplay between viral infection, tumor metabolism, and therapeutic outcomes. VSV strongly influences cellular metabolism during infection, not only to enhance viral replication but also to alter the tumor’s metabolic landscape, thereby affecting cancer progression and therapeutic response.

A recent study provided mechanistic insights into how VSV modulates tumor metabolism in GBM [76]. The authors focused on the VSV matrix (M) protein, a multifunctional viral factor responsible for host cell shutoff and immune evasion. They discovered that the VSV-M protein indirectly modulates mitochondrial function by promoting a metabolic shift toward aerobic glycolysis, similar to the “Warburg effect” observed in many cancers. This glycolytic reprogramming increased glucose uptake and lactate production, creating a metabolic environment favorable for VSV replication but also potentially supporting aggressive tumor phenotypes. Importantly, the metabolic rewiring induced by VSV rendered GBM cells susceptible to the glycolytic inhibitor 2-deoxyglucose (2-DG). Combination treatment with VSV and 2-DG synergistically enhanced tumor cell killing both in vitro and in vivo, demonstrating that metabolic vulnerabilities exposed by VSV infection can be therapeutically exploited. These findings highlight the potential of integrating metabolic inhibitors into OV therapy, not only to improve viral efficacy but also to prevent the tumor from leveraging virus-induced metabolic changes for its own survival and progression.

The metabolic effects of OVs are not limited to glycolytic pathways. In a complementary study, octyl itaconate, a derivative of the immunometabolite itaconate, was found to modulate cellular metabolism and antiviral signaling during VSV-ΔM51 infection [77]. The authors found that octyl itaconate enhanced VSV oncolytic activity by simultaneously targeting multiple metabolic and immune pathways. Specifically, octyl itaconate suppressed the expression of ISGs and inflammatory mediators by modulating redox balance and, subsequently, dampening NF-κB signaling. This metabolic modulation created a more permissive environment for viral replication while reducing pro-inflammatory responses that can limit viral spread or promote tumor growth. The study demonstrates how exogenous metabolic modulators can be strategically combined with OVs to optimize therapeutic efficacy, underscoring the intricate relationship between host cell metabolism and antiviral immunity.

Collectively, these studies highlight the dual nature of VSV-induced metabolic rewiring. On one hand, VSV hijacks tumor metabolic machinery to fuel its replication, potentially enhancing its oncolytic potency. On the other hand, these metabolic shifts may create niches that favor tumor adaptation, survival, or resistance if not properly targeted. Importantly, VSV’s ability to expose metabolic vulnerabilities, such as heightened glycolytic dependence, offers unique opportunities for therapeutic intervention. Combining VSV with metabolic inhibitors like 2-DG or metabolic modulators such as octyl itaconate may amplify tumor destruction while preventing unintended pro-tumorigenic effects.

### 5.7. VSV Encoding Vaccinia Virus Genes Evade Antiviral Immune Responses

As discussed above, pharmacological inhibitors, such as JAK/STAT pathway blockers, can be used to dampen the undesirable antiviral response in tumor cells, thereby transiently suppressing type I IFN signaling and reducing the expression of antiviral ISGs. While demonstrating effectiveness in many studies, this strategy has limitations, including potential systemic immunosuppression and off-target effects, which can raise safety concerns. As an alternative or complement to pharmacological inhibition, immune-evading mechanisms encoded by distinct viruses have been investigated. Among these, a large DNA virus, vaccinia virus (a poxvirus), encodes numerous proteins specifically designed to counteract host innate immune defenses. In a landmark study, Shors et al. examined the role of the vaccinia virus proteins E3L and K3L, both known IFN antagonists, in rescuing viruses from IFN-mediated inhibition [78]. Using cultured cells pretreated with IFN-α to simulate a restrictive antiviral state, they demonstrated that transient expression of E3L before VSV infection fully restored VSV replication, whereas K3L provided only partial protection. These experiments showed that E3L interferes with the host antiviral pathways that normally suppress VSV, providing proof of concept that engineering VSV with potent intracellular IFN suppressors could dramatically enhance its persistence and spread in otherwise nonpermissive environments. The work by Le Boeuf et al. demonstrated that vaccinia virus can alleviate innate immune barriers and enhance VSV-mediated tumor killing through the secretion of B18R, a soluble interferon (IFN) decoy receptor that sequesters type I IFNs and prevents them from activating antiviral signaling [79].

Building on these concepts, Maroun et al. evaluated whether co-administering VSV (VSV-mIFNβ-EGFP) with vaccinia virus, which naturally expresses E3L and B18R, could enhance VSV replication and therapeutic efficacy [80]. In this study, VSV alone was rapidly cleared in vivo due to its high sensitivity to IFN, whereas in combination with vaccinia virus, VSV replication was prolonged. When delivered together in mouse tumor models, vaccinia virus suppressed the local antiviral state through its IFN-modulating proteins, thereby creating a permissive environment for VSV replication. This “collateral effect” significantly increased VSV spread and tumor cell killing, resulting in superior tumor regression compared to either virus alone.

## 6. Increasing Direct Oncolysis by VSV

Direct oncotoxicity refers to the ability of VSV to kill infected cells independent of cell-mediated antitumor adaptive immunity (discussed later in this review). Various strategies to enhance direct cytotoxicity include (1) direct stimulation of cell death mechanisms, (2) VSV-encoded tumor suppressor genes, (3) combining VSV with standard chemotherapy, and (4) combining VSV with radiation therapy.

### 6.1. Direct Stimulation of Cell Death Mechanisms

Recent advances have focused on enhancing direct tumor cell killing while simultaneously promoting anti-tumor immunity. In a recent study, a recombinant VSV, VSV-S, that carries the SMAC/DIABLO gene inserted into its genome, enhanced programmed cell death in tumor cells. In this study, researchers evaluated its effects in head and neck squamous cell carcinoma (HNSCC) cell lines and in both immunocompromised and immunocompetent syngeneic mouse models of orthotopic HNSCC [81]. Compared to wt VSV, VSV-S induced substantially more apoptosis and more robust PANoptosis (a coordinated activation of pyroptosis, apoptosis, and necroptosis). These enhanced cell death types translated into more potent antitumor effects in vivo, with VSV-S more effectively reducing tumor volume, preventing lymph-node metastasis, increasing survival, and causing minimal systemic toxicity, compared to wt VSV. Significantly, VSV-S also boosted tumor infiltration of CD8^+^ T cells (including cytotoxic subsets) and induced chemokine expression (CXCL9, CXCL10), both of which are favorable for T cell trafficking. In addition, VSV-S treatment lowered PD-L1 expression on tumor cells and, when combined with anti-PD-1 immune checkpoint blockade, produced synergistic antitumor efficacy, greater tumor suppression, and survival benefit than either treatment alone [81]. Another study showed VSV-S enhanced apoptosis and anti-tumor immunity in triple-negative breast cancer (TNBC) models [82]. In an orthotopic TNBC mouse model, neoadjuvant VSV-S treatment of primary tumors significantly reduced lung metastasis following surgical excision and improved overall survival. Mechanistically, VSV-S remodels the immunosuppressive tumor microenvironment by decreasing myeloid-derived suppressor cells (MDSCs) and tumor-associated macrophages (TAMs), favoring neutrophil and CD8^+^ T cell infiltration, increasing IFN-γ, lowering TGF-β, and inducing apoptosis and potentiating the efficacy of immune checkpoint blockade [81,82]. These findings highlight a dual mechanism by which VSV-S amplifies both direct viral oncolysis and immune-mediated tumor clearance, providing a strong preclinical rationale for its use in next-generation oncolytic virotherapy, including in difficult-to-treat cancers such as TNBC and HNSCC.

### 6.2. VSVs Encoding Tumor Suppressor Genes

The strategic incorporation of tumor suppressor genes into VSV vectors has evolved from early foundational studies to more sophisticated, mechanism-based therapies. Initially, this approach was used by Heiber et al. to generate VSV recombinants expressing high levels of functional murine p53 (mp53) transgene inserted into the VSV genome [83,84,85]. VSV-mp53 was highly attenuated in vivo due to p53-activated innate immune genes but showed impressive efficacy in immunocompetent mice [83,84,85]. However, a challenge in using VSV encoding p53 is that p53 activation can induce antiviral responses in some tumor types with active type I IFN signaling, leading to VSV attenuation in tumor tissues and decreased oncolytic abilities of VSV. To examine this possibility, our study examined novel VSV-ΔM51-based recombinant viruses in PDAC cell lines encoding “wt” human p53 or the previously described chimeric p53-CC, which contains the coiled-coil (CC) domain from breakpoint cluster region (BCR) protein (evades the dominant-negative activities of endogenously expressed mutant p53) fused to a fluorescent reporter [83,84,85]. Contrary to an expected enhancement of antiviral signaling by p53, our study showed that both p53 and p53-CC inhibited type I IFN responses in cancer cells, but not in non-malignant human pancreatic ductal cells, which retained their resistance to all tested VSV recombinants. Our data suggested that the observed inhibition of type I IFN responses in tumor cells occurred through p53-mediated inhibition of the NF-κB pathway in PDAC cells [83,84,85]. VSV-p53 recombinants remain an auspicious approach for oncolytic virotherapy.

Another notable approach involves incorporating the XAF1 gene, a well-established tumor suppressor and pro-apoptotic gene, into VSV vectors. This modification has been shown to increase the antitumor efficacy of VSV by promoting cell death and enhancing antitumor immune responses [86]. Another study has explored the role of miRNAs in modulating p53’s tumor suppressor activity. This study indicated that p53 can transactivate tumor suppressor miRNAs or repress pro-cancer miRNAs (so-called oncomiRs), thereby boosting its tumor suppressor activity. This interplay suggests that integrating miRNA regulators into VSV vectors could further enhance their oncolytic properties [87].

### 6.3. Combining VSV with Standard Chemotherapy

The earliest studies combining VSV with chemical agents focused predominantly on conventional chemotherapeutics. Agents such as cisplatin and 5-fluorouracil (5-FU) were shown to suppress type I IFN responses in tumor cells, thereby facilitating VSV replication and enhancing oncolysis, particularly in breast, prostate, and melanoma cancer models [88,89,90]. These investigations established the principle that transient inhibition of intrinsic antiviral defenses could potentiate tumor cell killing while maintaining viral selectivity for malignant cells. Additionally, early work demonstrated that chemotherapeutic sensitization could extend VSV efficacy to otherwise resistant tumor types, highlighting the importance of understanding tumor-specific antiviral signaling in the design of combination strategies.

The synergy between VSV and chemotherapeutics has been further explored in a range of preclinical studies. Several studies have demonstrated that specific chemotherapeutic agents can directly sensitize tumors to VSV infection, thereby improving therapeutic outcomes. For example, combining VSV with low-dose cisplatin significantly enhanced antitumor efficacy against murine melanoma by promoting apoptosis and increasing viral spread within the tumor tissue [89]. Similarly, in squamous cell carcinoma models, concurrent administration of VSV and chemotherapy enhanced tumor regression compared to either treatment alone, underscoring the potential for synergistic killing of malignant cells [90]. Our study in PDAC cells showed that paclitaxel and other chemotherapy agents causing G2/M phase arrest markedly enhanced the replication of IFN-sensitive viruses such as VSV-ΔM51 and Sendai virus by suppressing type I and III interferon production and downstream antiviral gene expression [91]. This effect, absent in IFN-signaling-deficient cells, suggests that the global transcriptional repression during G2/M creates a transient vulnerability that viruses can exploit to promote replication [91]. Other approaches have leveraged VSV as a gene delivery platform, such as combining VSV with a cytosine deaminase suicide gene, which further augmented tumor cell killing when paired with prodrug treatment [88]. Expanding beyond traditional chemotherapies, innovative strategies like pairing VSV with photodynamic therapy have shown promise, as demonstrated in glioblastoma models where a VSV–porphyrin combination provided potent tumor clearance through dual mechanisms of phototoxic and oncolytic activity [92].

However, the relationship between chemotherapy and VSV is not universally synergistic. Emerging evidence indicates that acquired chemoresistance can confer cross-resistance to VSV, limiting the efficacy of combination regimens. In PDAC, experimentally acquired chemoresistance of PDAC cells resulted in increased resistance to VSV, suggesting that shared pathways govern sensitivity to both modalities [93]. Similarly, drug-resistant melanoma cells lacking SOX10 were found to be refractory to multiple oncolytic RNA viruses, including VSV, highlighting the challenge of targeting tumors that evolve broadly resistant phenotypes [94]. Moreover, specific chemotherapeutic agents may directly inhibit VSV replication, potentially antagonizing its oncolytic activity. For instance, pralatrexate, an antifolate used in lymphoma treatment, was recently shown to suppress VSV replication in vitro, emphasizing the need for careful selection and timing of drug-virus combinations to avoid interference [95].

Collectively, these findings illustrate the nuanced interplay between VSV and chemotherapeutics. While many conventional agents can enhance VSV-mediated tumor killing by weakening antiviral defenses, altering the cell cycle, or providing synergistic cytotoxic stress, others may induce resistance or directly inhibit viral replication. Moving forward, rationally designed combinations guided by mechanistic insights and patient-specific tumor profiles hold the most significant promise for maximizing VSV’s therapeutic index while minimizing antagonistic effects.

### 6.4. Combining VSV with Radiation Therapy

Combining oncolytic VSV with radiation therapy represents a promising strategy to enhance tumor cell killing and stimulate systemic antitumor immunity. Radiation can modulate the tumor microenvironment by inducing DNA damage, stress responses, and localized immunogenic cell death. Still, it also transiently suppresses antiviral signaling in tumor cells, creating a window in which VSV can replicate more efficiently. This principle was demonstrated in a study using VSV expressing IFNβ in prostate cancer models, where radiation attenuated tumor antiviral responses, allowing robust viral replication and oncolysis. The combination treatment led not only to direct tumor cell killing but also to pronounced systemic immune responses, including increased activation of cytotoxic T cells and improved antitumor immunity at distant sites [96].

To date, this appears to be the only published study since 2012 [97] directly combining VSV with radiation therapy, which may reflect the experimental complexity of synchronizing viral delivery with precise radiation timing, concerns about toxicity, and the need for tumor models that accurately recapitulate both immune and viral responses. Potential limitations include the risk of enhanced inflammation or tissue damage in normal tissues due to combined viral replication and radiation-induced stress, as well as variability in tumor sensitivity to both modalities. Nevertheless, these findings suggest that radiation can act as a sensitizer for VSV therapy, highlighting a rational framework for future preclinical and translational studies.

## 7. Improving Delivery of VSV to the Tumor and Strategies to Prevent Premature Clearance of VSV by the Immune System

### 7.1. Physical/Chemical Carriers

A significant limitation of VSV-based oncolytic therapy is the premature clearance of the virus by the host immune system, particularly by neutralizing antibodies and intravascular immune cells, which can restrict viral dissemination and limit therapeutic efficacy. In addition, VSV and VSV-G-pseudotyped vectors can be inactivated by human serum complement even in the absence of pre-existing immunity to VSV [98,99]. To overcome these issues, multiple strategies have been explored to enhance tumor delivery and protect the virus during systemic circulation.

Physical and carrier-based delivery methods aim to shield VSV from immune detection and facilitate targeted tumor delivery [100]. Techniques such as liposomal encapsulation, polymeric nanoparticles, and cell-based delivery systems have demonstrated stability and tumor targeting. These carriers can shield the virus from immune surveillance and facilitate targeted delivery to tumor sites.

### 7.2. Cellular Carriers

Cell-based carriers use a “Trojan horse” mechanism, in which the virus is transported within cells to evade immune detection. T cells offer a unique advantage in cancer therapy, not only through their intrinsic cytotoxic activity against malignant cells but also by orchestrating broader immune responses by recruiting additional effector cells [101]. Their ability to recognize tumor-associated antigens further enhances their precision in targeting cancerous tissues [102]. Adoptive T cell therapies, in which effector T cells are expanded ex vivo, have emerged as a promising strategy, with peripheral blood providing a readily accessible source from which mononuclear cells can be isolated via density gradient centrifugation. These cells are then stimulated and expanded using tailored cytokine cocktails, producing sufficient numbers for therapeutic use [103]. Importantly, this approach can synergize effectively with OVs. For example, Qiao et al. demonstrated that autologous naïve T cells can serve as carriers for VSV, facilitating the elimination of metastatic cells in lymphoid tissues [104]. By incubating T lymphocytes with VSV, the virus attaches to the cell surface and is subsequently delivered to tumor sites. Within 72–96 h, as the T cells migrate to the tumor, the virus is released, enabling infection of tumor cells and lymph nodes and enhancing the efficacy of viral oncolysis [104].

Other carrier cell types, particularly mesenchymal stem cells (MSCs), have been leveraged for VSV delivery. MSCs can be efficiently transduced with VSV or VSV-G-pseudotyped lentiviral vectors, enabling prolonged viral carriage and targeted delivery to tumors [105,106]. Directed fusion of MSCs with target cells via VSV-G further facilitates functional viral delivery and can improve therapeutic outcomes in solid tumor models. Repeated viral dosing has also been shown to enhance efficacy, in part through interactions with intravascular monocytes that aid in viral distribution [107].

### 7.3. VSV-Encoded Transgenes Preventing Rapid Virus Elimination

Viral engineering strategies provide complementary approaches to reduce premature clearance. For example, modifications such as the expression of immunostimulatory cytokines like interleukin-12 in VSV-GP reshape the immune response to favor durable CD8^+^ T-cell–mediated antitumor activity rather than rapid virus elimination [108,109].

## 8. Enhancing VSV-Mediated Stimulation of Tumor-Specific Immunity

### 8.1. Combining VSV with Immunomodulation to Stimulate Tumor-Specific Immunity: General Considerations

VSV’s natural ability to induce tumor-specific immunity is a key aspect of its oncolytic activity. The virus can stimulate both innate and adaptive immune responses, leading to the activation of cytotoxic T lymphocytes and the production of pro-inflammatory cytokines. Immunomodulation can also be achieved by using certain VSV monotherapies (Table 2), most notably VSV-IFNβ-NIS, which demonstrated enhanced immune engagement, functional imaging capabilities, as well as compatibility with radiotherapy in both solid and hematologic malignancies (Clinical Trial NCT03647163). Cytokine therapies and other immune modulators can be combined with VSV to enhance both direct tumor cell killing and durable antitumor immune responses. For example, cytokines and immune modulators, including IL-2 and IFN-α, synergized with VSV to enhance both direct tumor cell killing and subsequent antitumor immune responses [110]. Similarly, a VSV-Δ51M strain expressing IL-12 demonstrated enhanced antitumor immune activation, increasing cytotoxic T cell infiltration and proinflammatory cytokine production in preclinical cancer models [31].

By expressing tumor-associated antigens or neoantigens, VSV can enhance the specificity of the immune response against cancer cells. A study engineered a recombinant VSV^M51R-Neo−2/15^ by inserting the Neo-2/15 IL-2 mimic to enhance its immunostimulatory capacity [111]. Arming VSV with Neo-2/15, a synthetic IL-2 mimic, enabled the virus to overcome the immunosuppressive TME. IL-2 is a key cytokine for activating and expanding cytotoxic CD8^+^ T cells and NK cells, but systemic administration is highly toxic. By encoding Neo-2/15 directly into the virus, the cytokine is produced locally within tumors, thereby promoting T-cell activation precisely where needed while minimizing systemic side effects. Importantly, when combined with anti-PD-L1 therapy, VSV^M51R-Neo−2/15^ produced synergistic antitumor effects, resulting in improved tumor regression, survival, and the induction of long-term antitumor immunity. Building on this approach, VSV engineered to express IL-2 or IL-2 mimics has been shown to act as a potent adjuvant for cancer vaccination, locally stimulating T and NK cells while avoiding the systemic toxicity of recombinant cytokines (Clinical Trial NCT02285816) [110].

**Table 2:** Table of VSV recombinants involved in immunomodulation.

VSV Variant	Cancer Target	Model	Key Findings	Transgene/Combination	Reference/s
VSV-interleukin-2/VSV-mIL-2	Melanoma	Mice	Interleukin-2 has improved vaccination adjuvant potential.	Mouse Interleukin-2	[110]
VSV M51R-mIL-2	Colon and lung	Mice	Interleukin-2 has improved vaccination adjuvant potential, with an attenuated form of VSV	Mouse Interleukin-2	[111]
VSV-mIL12-mGMCSF	Melanoma	Mice	Partial inhibition of tumor growth in mice bearing B16-F10 melanoma tumors.	Cytokine interleukin-2 (IL-2), and murine Granulocyte-Macrophage Colony-Stimulating Factor (mGM-CSF)	[112]
VSV M51R-Neo-2/15	Colon and lung	Mice	Boost anti-tumor immunity without the toxicity linked to traditional IL-2 therapies.	De novo synthesized cytokine that functions as both IL-2 and IL-15	[111]
VSVΔM51-IL-15	Pancreatic	Mice	Found to enhance tumor regression and increase survival time when combined with natural killer T (NKT) cell activation therapy.	Cytokine IL-15	[113]
VSV-IFNß/VSV-hIFNβ	Hepatocellular carcinoma, prostate, lung, melanoma, NSCLC	Mice	Increasing tumor selectivity, protecting normal cells, and boosting anti-tumor immune responses.	Human interferon-ß	[58,72,96,114,115,116]
VSV-mIFNβ	Melanoma	Mice	Increasing tumor selectivity, protecting normal cells, and boosting anti-tumor immune responses.	expresses mouse interferon-ß	[117]
VSV-IFNβ-NIS	Osteosarcoma, lymphomas, melanoma, lung, anal adenocarcinoma, myeloma	Dogs & Pigs	Increasing tumor selectivity, protecting normal cells, and boosting anti-tumor immune responses.	expresses human interferon-ß, as well as a reporter (NIS)	[118,119,120]
VSV-mIFNβ-NIS	Myeloma and colon	Mice	increasing tumor selectivity, protecting normal cells, and boosting anti-tumor immune responses.	expresses mouse interferon-ß, as well as a reporter (NIS)	[121]
VSV-IFNß-Lcn2	Hepatocellular carcinoma	Mice	This recombinant was able to refocus immunity toward tumor-specific T cells.	Expresses human IFNß, while also expressing lipocalin 2 (Lcn2), a small, secreted glycoprotein.	[116]
VSV-IFN-β, -CSDE1WT or -CSDE1C−T	Melanoma	Mice	Forming a “trap and ambush” strategy that enhances tumor control and sensitizes resistant tumors to immune checkpoint blockade.	Expresses human IFNß, while also expressing Cold Shock Domain Containing E1 (CSDE1)	[60]
VSV-IFNß-Lect2	Hepatocellular carcinoma	Mice	VSV expressing tumor antigens, restoring synergy with checkpoint blockade for anti-PD-L1 therapy.	Expressing human IFNß, while also expressing LECT2 (leucocyte cell-derived chemotaxin 2)	[116]
VSV-IFNß-Smagp	Hepatocellular carcinoma	Mice	VSV expressing IFNβ and SMAGP enhanced tumor-selective oncolysis and antitumor immune responses, improving survival compared with the parental VSV-IFNβ.	Expresses human IFNß, and SMAGP	[116]
VSV-CXCL9	Lung	Mice	Further boosting of the functional chemokine gradient.	Expresses the chemokine CXCL9	[122]
VSV-Δ51M-hIL-12	Melanoma	Mice and co-culture	Uses IL-12 to trigger apoptosis in tumor cells, as well as IFN-γ induction, which triggers.	Expresses Human Interleukin-12 in the form of attenuated VSV	[31]
VSV-mCSDE1^WT^	Melanoma	Mice	Assisted in sifting the tumor microenvironment to make it more susceptible to OV.	Mouse Cold Shock Domain-containing E1 protein (mCSDE1)	[117]

### 8.2. Co-Administration of VSV with ICI

A significant challenge in oncolytic virotherapy is that while viral infection can generate immunogenic cell death and recruit immune effector cells, the resulting antitumor immune response is often blunted by T cell exhaustion and immune checkpoint upregulation within the tumor microenvironment. Combining OVs with ICIs, including anti-PD-1, anti-PD-L1, and anti-CTLA-4 antibodies, further amplifies T cell-mediated tumor clearance by exploiting VSV-induced immunogenic cell death, improving therapeutic outcomes [43,59,123].

A notable example is a chimeric VSV–Newcastle disease virus (VSV-NDV) evaluated in a murine melanoma model [43]. This recombinant virus induced strong activation of the RIG-I signaling pathway, enhancing type I interferon production and T cell recruitment. When combined with anti-CTLA-4 checkpoint blockade, the therapy produced synergistic tumor regression, demonstrating how VSV’s innate immune activation can be leveraged to boost the efficacy of ICIs. Similarly, the role of additional immune checkpoints has been highlighted by work examining T cell immunoglobulin and mucin-domain containing-3 (Tim-3). Peripheral delivery of Tim-3-blocking antibodies reduced VSV-associated neurotoxicity by enhancing MHC class I antigen presentation [124]. In a separate mechanistic study, Tim-3 signaling was shown to facilitate viral infection by suppressing the USP25-TRAF3-IRF7 pathway [125]. These findings suggest that targeting Tim-3 may simultaneously improve both safety and efficacy of VSV therapies. Another study showed that combining IL-15-expressing VSV with Natural Killer T (NKT) cell activation and PD-1 blockade produce robust antitumor effects in PDAC models [113]. The therapy enhanced infiltration and activation of NKT, NK, and CD8^+^ T cells, leading to significant tumor regression, prolonged survival, and the induction of immune memory. ICIs amplify the immunogenic effects of VSV-induced tumor cell death, improving antigen presentation and generating a positive feedback loop that sustains viral propagation within the tumor. Studies have demonstrated that this combination can lead to durable remissions in patients with various malignancies [126,127].

Significantly, tumor genetics can influence responses to combination therapy. In melanoma, mutations in the IFNγ-JAK-STAT pathway drive resistance to checkpoint inhibitors but, paradoxically, increase tumor sensitivity to oncolytic VSV treatment [128]. This indicates that OVs may provide an alternative therapeutic option for patients who fail ICI therapy, and that molecular profiling of tumors could help tailor combination regimens.

### 8.3. Combining VSV-Based Oncolytic Virotherapy with Anti-Cancer Vaccines

VSV-based oncolytic virotherapy has emerged as a versatile platform not only for direct oncolysis but also for anti-cancer vaccination, leveraging its ability to induce immunogenic cell death, release tumor-associated antigens (TAAs), and prime adaptive immune responses. Preclinical studies across multiple cancer types have demonstrated that VSV-based vaccines can reshape the TME, enhance DC activation, and drive robust CD8+ T cell responses.

Several studies highlight the synergistic potential of combining oncolytic VSV-based therapy with tumor vaccines. Administering VSV alongside peptide- or protein-based tumor vaccines in murine models significantly enhanced antitumor efficacy compared to either vaccine monotherapy [37,129]. The combination modulated the TME, reducing immunosuppressive elements and increasing infiltration and activation of CD8^+^ T cells in subcutaneous and orthotopic models targeting melanoma and colon carcinoma, with combination therapy yielding enhanced tumor regression and survival.

Another study demonstrated that modular, self-adjuvanted cancer vaccines, when paired with VSV vectors, elicited potent antitumor immunity [36]. These vaccines incorporated toll-like receptor (TLR) agonists or other immune stimulatory motifs into the peptide vaccine, which, when combined with VSV, led to enhanced DC maturation, higher CD8^+^ T-cell activation, and improved tumor control. Similarly, heterologous prime-boost strategies in which peptide-based vaccines were followed by VSV vectors expressing the same or related tumor antigens reshaped DC and T-cell phenotypes, enhanced effector/memory responses, and improved tumor eradication in models of melanoma and breast cancer [130].

Several studies explored antigen-specific VSV engineering to enhance vaccine efficacy. An example showing VSV-G glycoprotein can be modified to express Her2/Neu antigens, allowing selective targeting of large mammary tumors in murine models, inducing strong anti-Her2 CD8^+^ T cell responses and complete tumor regression [24]. Another study evaluated VSV-GP encoding HPV antigens, demonstrating robust cytotoxic T-cell induction and tumor suppression in cervical cancer models [40]. Additionally, encoding destabilized tumor antigens in VSV vectors improved antigen processing and presentation, increasing activation of antigen-specific CD8^+^ T cells and improving antitumor efficacy [131]. Neoantigen-targeted approaches showed that VSV-induced neoantigen formation initially restricted viral replication but could be overcome by combination immunotherapy, highlighting VSV’s capacity to both induce and expose novel tumor antigens for immune targeting [117].

Antiviral and vaccine-specific CD8^+^ T-cell populations have been characterized after VSV-GP-based cancer vaccination, demonstrating durable antigen-specific T-cell memory and systemic antitumor immunity [34]. Immune modulation studies also show that checkpoint blockade can enhance the efficacy of the VSV vaccine. For example, it was demonstrated that NKG2A blockade combined with cancer vaccines had a bimodal effect on i.t. and systemic CD8^+^ T-cell responses, underscoring the potential of checkpoint modulation to enhance VSV-induced antitumor immunity further [34]. Across multiple studies, VSV-based vaccination consistently altered TME composition, increased effector T-cell infiltration, reduced suppressive populations (including Regulatory T cells (Tregs) and MDSCs), and enhanced DC maturation, collectively supporting more potent and durable antitumor responses.

Despite these advancements, several challenges remain in the clinical application of VSV-based neoantigen vaccines. The identification and validation of suitable neoantigens are complex and resource-intensive processes. One major challenge is eliciting strong tumor-specific immunity without being dominated by antiviral responses, a problem the authors metaphorically describe as “finding the tumor antigen needle in the antiviral haystack” [132]. High viral immunogenicity can divert immune responses toward viral antigens rather than tumor-associated antigens, and tumor antigen heterogeneity can further limit efficacy. In contrast, the immunosuppressive tumor microenvironment can hinder the effectiveness of these therapies.

### 8.4. Leveraging VSV to Enhance CAR T Therapy

Chimeric antigen receptor (CAR) T cell therapy is an adoptive immunotherapy in which patient-derived T cells are genetically engineered to recognize and kill tumor cells in an antigen-specific manner. While highly effective in certain hematologic malignancies, CAR T therapy faces significant challenges in solid tumors, including limited T cell expansion, poor persistence, and insufficient infiltration within the immunosuppressive tumor microenvironment. Oncolytic VSV has emerged as a versatile tool to overcome these barriers by enhancing both CAR T cell function and tumor-targeted immune responses. Preclinical studies have demonstrated that OVs can be used to expand dual-specific CAR T cells, improving their proliferation and antitumor efficacy in murine solid tumor models [133]. Beyond systemic viral exposure, loading CAR T cells with oncolytic VSV has been shown to directly enhance CAR-mediated signaling, boost cytotoxic activity, and improve tumor clearance in vivo [134].

## 9. Expanding the Vesiculovirus Toolbox: Oncolytic Features of Maraba, Morreton, and Jurona Viruses

Maraba virus, Morreton virus and Jurona virus are rhabdoviruses closely related to VSV and belonging to the same genus Vesiculovirus. Maraba virus has shown potent oncolytic activity and is currently being investigated in clinical trials. Morreton virus and Jurona virus have also demonstrated good oncolytic potential in preclinical studies. The development of these viruses as oncolytic agents offers additional options for oncolytic virotherapy and may provide alternative therapies for patients with cancers resistant to existing treatments.

The foundational study established the attenuated Maraba MG1 strain as a potent platform for an “oncolytic vaccine vector”, demonstrating its ability to both lyse tumor cells and elicit robust tumor antigen-specific immune responses [135]. In murine B16-F10 melanoma models, including lung metastases and intracranial tumors, MG1 engineered to express human dopachrome tautomerase (MG1-hDCT) effectively infected and killed tumor cells and preferentially replicated in tumor-bearing tissues. Although MG1-hDCT alone had a limited therapeutic effect, it provided proof of concept that MG1 could serve as a tumor-targeted oncolytic platform. Subsequent studies further demonstrated that MG1 monotherapy was highly effective in small tumors (~90% cure rate) but less so in larger, immunologically “cold” tumors [136].

MG1-based vaccination robustly activated tumor-specific immunity. In ovarian cancer models, a Maraba-OVA (MRB-OVA) boost following peptide/adjuvant priming elicited potent tumor-infiltrating CD8^+^ T cell responses and delayed progression in an intraperitoneal ovarian tumor model [137]. However, therapeutic efficacy was limited by T-cell dysfunction in the tumor microenvironment. The addition of PD-1 blockade restored T cell activity, enhanced survival, and produced gene signatures predictive of improved outcomes in human ovarian cancer datasets. Similarly, in advanced, immunologically “cold” melanoma, MG1 combined with anti-PD-1 therapy partially reversed immune suppression, enhanced CD8^+^ T cell responses, and extended survival compared with monotherapy (~77.5 vs. ~41–46 days) [136].

Translational relevance was bolstered by a feline safety study by Hummel et al., which tested the adenovirus-prime/MG1-boost strategy in healthy cats using tumor-associated antigens huDCT and huPLAC1 [138]. Across escalating MG1 doses up to 2.5 × 10^11^ pfu, the regimen was well-tolerated, with only transient side effects and no evidence of viral shedding or tissue pathology. These findings confirmed the platform’s safety in a large-animal model and paved the way for veterinary and possibly comparative oncology trials.

Morreton virus (MORV) shares many structural and genomic similarities with VSV but possesses distinct biological properties that may offer therapeutic advantages. A key advantage of MORV over VSV is its reduced neurovirulence, a critical safety consideration given VSV’s inherent neurotropism that can complicate systemic delivery [139]. MORV demonstrated efficient infection and lysis of HCC cells in vitro and potent antitumor efficacy in vivo [139]. Building on these findings, a chimeric vesiculovirus was generated by replacing the VSV glycoprotein with the MORV glycoprotein [44]. This hybrid virus demonstrated multi-modal efficacy against sarcomas, combining the robust replication and cytolytic activity of the VSV backbone with the distinct cell entry and immune-modulating properties conferred by the MORV glycoprotein. The MORV glycoprotein broadened viral tropism, enabling infection of sarcoma cells otherwise resistant to VSV, thereby extending the reach of vesiculovirus-based therapies.

Despite these advances, tumor-intrinsic factors can still limit MORV efficacy. A recent study systematically profiled PDAC to identify resistance signatures shared across oncolytic vesiculoviruses, including VSV and MORV [140]. The authors found that PDAC cells exhibiting high basal IFN signaling and upregulation of innate antiviral effectors were cross-resistant to both viruses, underscoring the challenge posed by tumors with robust intrinsic antiviral states. These findings emphasize that, while MORV can bypass certain VSV-specific limitations, it remains vulnerable to similar innate immune barriers, necessitating combination strategies such as immune modulators or chemotherapy to enhance viral replication and spread within the tumor.

A recent study introduced Jurona virus (JURV) as a novel vesiculovirus with strong potential for oncolytic virotherapy in HCC [141]. JURV demonstrated potent cytolytic activity across multiple human and murine HCC cell lines, with rapid replication kinetics and a favorable safety profile, showing minimal neurotoxicity even at high systemic doses. In both xenograft and syngeneic mouse models, i.t. JURV treatment significantly reduced tumor growth and even produced abscopal effects, with uninjected tumors exhibiting delayed progression, suggesting systemic antitumor activity. Importantly, JURV exhibits low pre-existing seroprevalence in humans and markedly reduced neurovirulence compared to VSV, supporting its suitability for systemic delivery and combination immunotherapy approaches in liver cancer.

While VSV remains the most extensively studied vesiculovirus for OV, its clinical translation is challenged by limitations, including rapid clearance by immune system and dose-limiting neurotoxicity. Other vesiculoviruses might offer distinct advantages across safety, tropism, immunogenicity, and translational potential (Table 3). MG1 and MORV exhibit reduced neurovirulence compared to VSV, enhancing their suitability for systemic delivery. In addition, unlike VSV, the MG1 is resistant to complement in non-immune human serum [98]. JURV also demonstrates a favorable safety profile, showing minimal neurotoxicity even at high doses in vivo, although a broader array of studies remains to be done. MORV’s unique glycoprotein confers distinct receptor usage and expanded tropism, enabling infection of tumor types resistant to VSV and improving tumor targeting in sensitive tissues such as the liver. These alternative vesiculoviruses offer complementary strengths to VSV, enabling the tailoring of oncolytic strategies based on tumor type, patient immune status, and therapeutic goals.

**Table 3:** Comparative properties of vesiculoviruses for oncolytic virotherapy.

Virus	Key Features	Safety	Tropism	Immunogenicity	Clinical Potential	Advantages over VSV
Vesicular stomatitis virus (VSV)	Prototype vesiculovirus; fast replication and potent oncolysis; extensively studied.	Moderate neurovirulence (limits systemic dosing).	Broad tropism but restricted by antiviral state.	Strong innate activation; moderate adaptive priming.	Most developed platform (multiple recombinant and clinical variants).	Benchmark platform; highest validation.
Maraba virus (MG1)	Attenuated, complement-resistant strain; effective in prime-boost vaccination.	Reduced neurovirulence; well-tolerated in animal models.	Like VSV, it infects a range of tumors.	Highly immunogenic, potent CD8^+^ T-cell priming in prime-boost regimens.	Clinical trials ongoing (vaccine and oncolytic applications).	Lower seroprevalence, complement resistance, and enhanced vaccine efficacy.
Morreton virus (MORV)	Naturally attenuated vesiculovirus; distinct glycoprotein receptor usage.	Safer than VSV; markedly reduced neurovirulence.	Broader tropism due to MORV-G; infects VSV-resistant cells.	Comparable innate activation; adaptive potential under study.	Preclinical efficacy in liver and sarcoma models.	Safer systemic use and enhanced tumor targeting.
Jurona virus (JURV)	Novel vesiculovirus; low human seroprevalence; rapid replication.	Minimal neurotoxicity even at high doses.	Preferentially targets hepatocellular carcinoma; potential broader use.	Potent immune remodeling, CD8^+^ infiltration, IFN-γ, PD-1 induction.	Early-stage, promising preclinical efficacy in HCC; synergizes with PD-1 blockade.	Strong systemic safety, immune activation, and low pre-existing immunity.

## 10. Conclusions

### 10.1. Current Challenges

**Figure 2:**
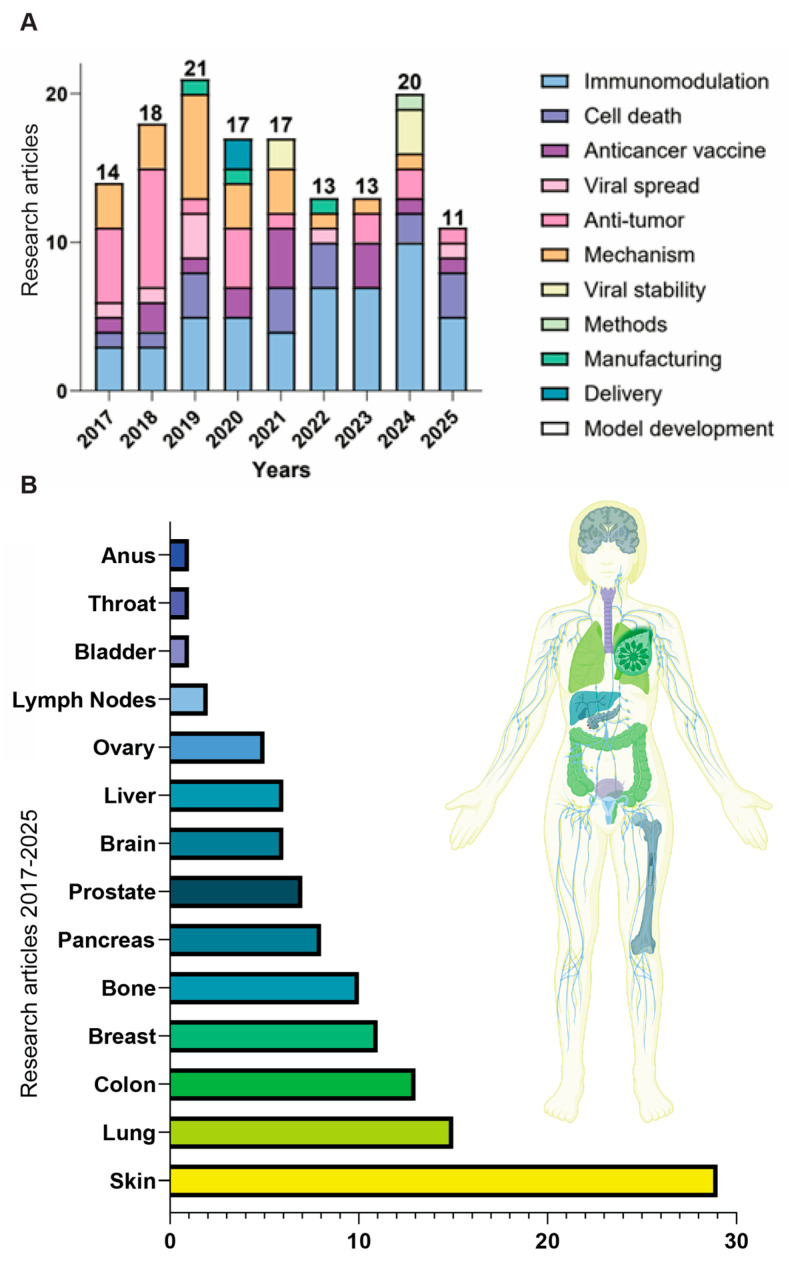
(**A**) Publication focuses of oncolytic VSV from 2017–2025. This stacked bar graph depicts the annual distribution of research publications using VSV by primary focus areas, including immunomodulation, induction of cell death, anticancer vaccine development, viral spread, direct antitumor activity, mechanistic studies, viral stability, methodological advancements, manufacturing, delivery strategies, and preclinical model development. Each bar represents the total number of publications each year, with color-coded segments indicating the proportion of studies in each research category. (**B**) Distribution of cancer types studied with oncolytic VSV in published research from 2017–2025. Literature was retrieved using the keywords “VSV” and “oncolytic”.

VSV-based oncolytic virotherapy has evolved from foundational studies toward sophisticated translational applications, integrative approaches such as immune modulation, targeted delivery, combination with chemotherapeutics or immunotherapies, and other treatments (Figure 2A, Table 1). This transition reflects the field’s maturation from basic scientific exploration to tackling clinically relevant hurdles, such as tumor selectivity, antitumor immunity, and the tumor microenvironment. Since 2017, oncolytic VSV has been explored in a range of malignancies, showcasing its broad applicability as a cancer therapeutic (Figure 2B).

Despite impressive progress, the field of VSV-based oncolytic virotherapy now stands at a pivotal juncture. Efforts to improve safety through VSV-based chimeric OVs, IFN-arming, microRNA targeting, and rational attenuation have undoubtedly reduced the risks of neurotoxicity and off-tumor replication. Yet these innovations raise a fundamental question: how much safety can be engineered without compromising the virus’s therapeutic potency? The field continues to debate whether maximal oncoselectivity is truly optimal, as sufficient infection of stromal and immune cells may be necessary to induce the type of inflammatory signaling that underpins durable systemic antitumor immunity.

Equally unresolved is the strategic identity of VSV as a therapeutic. Should it be advanced primarily as a lytic monotherapy, optimized for direct tumor cell killing? Or should its greatest value lie in functioning as an adjuvant to immune checkpoint blockade, where its ability to inflame the tumor microenvironment may prove more important than its oncolytic capacity? A third vision sees VSV as a versatile delivery chassis, optimized less for oncolysis per se and more for transporting payloads, cytokines, co-stimulatory ligands, bispecific engagers, or even imaging tools, into tumors. These divergent trajectories reflect different philosophies of what oncolytic virotherapy should achieve and complicate the field’s long-term direction.

Another pressing debate surrounds systemic delivery. It remains unclear whether progressive refinements to the viral backbone, attenuating mutations, gene-order rearrangements, chimeric envelopes, or microRNA-regulated expression, will be sufficient to allow safe intravenous administration. Some groups propose that even the most attenuated VSVs may still require transient immunosuppression to permit effective systemic spread. This strategy reintroduces clinical risks and may blunt the very immune responses that make VSV attractive as cancer immunotherapy.

Ultimately, the rapid evolution of synthetic virology poses a significant regulatory challenge. VSV is increasingly being developed as a platform technology, with modular backbones that can accept interchangeable payloads or targeting motifs. This creates the potential for “libraries” of VSV variants to be generated quickly and tested in parallel. However, current regulatory frameworks are designed to evaluate fixed, single-product biologics rather than rapidly iterated, modular platforms. Should each engineered VSV variant be treated as a new therapeutic requiring complete preclinical and clinical evaluation, or should approval be streamlined once the safety of a backbone is established? The answer will have profound implications for how quickly these therapies can advance from bench to bedside.

### 10.2. Future Directions

Looking forward, continued development of VSV-based therapies will likely focus on precision-guided strategies that combine insights from tumor-specific antiviral signaling, immunological profiling, and high-throughput preclinical screening. As the field advances, these efforts are expected to expand the clinical applicability of VSV-based oncolytic virotherapy across a broader spectrum of malignancies and bring the potential for personalized, highly effective viral anti-cancer treatments closer to reality.

The trajectory of VSV-based virotherapy will likely be shaped as much by these translational and regulatory decisions as by technical innovation itself. The field must confront uncomfortable but essential questions about trade-offs between potency and safety, the desired role of VSV in the therapeutic landscape, and the regulatory models best suited for a modular, rapidly evolving platform. Ultimately, the future of VSV may depend not only on what is scientifically achievable but also on what is judged clinically feasible, ethically acceptable, and societally valuable.

## Data Availability

Not applicable.

## Supplementary Materials

The following supporting information can be downloaded at: https://www.mdpi.com/article/10.3390/curroncol32110627/s1, Supplemental Table S1. Comprehensive list of recombinant VSV constructs reported since 2017, including details on genetic modifications, targeted mechanisms of action, therapeutic applications, and associated cancer models. This table highlights advancements in VSV engineering aimed at improving oncoselectivity, safety, neurotoxicity, VSV efficacy, and immune modulation of the tumor microenvironment.

## Abbreviations

The following abbreviations are used in this manuscript:

CAR	chimeric antigen receptor
CD200AR-L	CD200 activation receptor ligand
CSDE1	Cold Shock Domain-containing E1
CTLA-4	cytotoxic T-lymphocyte-associated protein 4
DC	dendritic cell
EV	programmed extracellular vesicle
F	fusion protein
GMB	glioblastoma
HCC	hepatocellular carcinoma
HDACi	histone deacetylase inhibitors
HN	hemagglutinin–neuraminidase
HNSCC	head and neck squamous cell carcinoma
ICI	immune checkpoint inhibitor
IFN	interferon
ISG	interferon-stimulated gene
i.t.	intratumoral
JAK	Janus kinase
JURV	Jurona virus
LASV-VSV	VSV encoding glycoprotein from Lassa virus
LDL-R	low-density lipoprotein receptor
MDSCs	myeloid-derived suppressor cells
miRNA	microRNA
MORV	Morreton virus
MSCs	mesenchymal stem cells
NDV	Newcastle disease virus
NIS	sodium iodine symporter
NK cell	Natural Killer cell
NKT cell	Natural Killer T cell
NSCLC	non-small cell lung cancer
OV	oncolytic viruses
PD-1	programmed cell death protein 1
PD-L1	programmed cell death ligand 1
RIG-I	Retinoic acid-inducible gene I
SIRT1	Sirtuin 1
STAT	signal transducer and activator of transcription
TACE	transarterial chemoembolization
TAMs	tumor-associated macrophages
TARE	radioembolization
TLR	toll-like receptor
TME	tumor microenvironment
TNBC	triple-negative breast cancer
Tregs	Regulatory T cells
VLP	virus-like particle
VMG	VSV encoding glycoprotein from Morreton virus (MorV)
VEGF	vascular endothelial growth factor
VSV	vesicular stomatitis virus
VSV-EBOV	VSV encoding glycoprotein from Ebola virus
wt	wild-type
